# Positron emission tomography-computed tomography in the diagnostic evaluation of smoldering multiple myeloma: identification of patients needing therapy

**DOI:** 10.1038/bcj.2015.87

**Published:** 2015-10-23

**Authors:** B Siontis, S Kumar, A Dispenzieri, M T Drake, M Q Lacy, F Buadi, D Dingli, P Kapoor, W Gonsalves, M A Gertz, S V Rajkumar

**Affiliations:** 1 Department of Internal Medicine, Mayo Clinic, Rochester, MN, USA; 2 Division of Hematology, Mayo Clinic, Rochester, MN, USA; 3 Division of Endocrinology, Mayo Clinic, Rochester, MN, USA

## Abstract

We studied 188 patients with a suspected smoldering multiple myeloma (MM) who had undergone a positron emission tomography-computed tomography (PET-CT) scan as part of their clinical evaluation. PET-CT was positive (clinical radiologist interpretation of increased bone uptake and/or evidence of lytic bone destruction) in 74 patients and negative in 114 patients. Of these, 25 patients with a positive PET-CT and 97 patients with a negative PET-CT were observed without therapy and formed the study cohort (*n*=122). The probability of progression to MM within 2 years was 75% in patients with a positive PET-CT observed without therapy compared with 30% in patients with a negative PET-CT; median time to progression was 21 months versus 60 months, respectively, *P*=0.0008. Of 25 patients with a positive PET-CT, the probability of progression was 87% at 2 years in those with evidence of underlying osteolysis (*n*=16) and 61% in patients with abnormal PET-CT uptake but no evidence of osteolysis (*n*=9). Patients with positive PET-CT and evidence of underlying osteolysis have a high risk of progression to MM within 2 years when observed without therapy. These observations support recent changes to imaging requirements in the International Myeloma Working Group updated diagnostic criteria for MM.

## Introduction

Smoldering multiple myeloma (SMM) is an intermediate stage between monoclonal gammopathy of undetermined significance and active MM.^1^ The risk of progression to active malignancy in the first 5 years of diagnosis is ~50%.^2^ According to International Myeloma Working Group (IMWG) criteria, the diagnosis of SMM requires clonal bone marrow plasma cells (BMPC) of 10–60% and/or a serum monoclonal (M) protein >3 g/dl, plus the absence of hypercalcemia, renal failure, anemia and bony lesions (CRAB features) or other myeloma defining events.^3^ The current standard of care is observation without therapy until development of symptoms^4^. Early treatment as opposed to watchful waiting of patients with highest risk of progression has the potential to improve progression-free and overall survival.^5^ The development of biomarkers to aid in distinguishing these high-risk patients is an area of active interest, and has included assessment of BMPC percentage, serum M protein, serum-free light-chain ratio and immunophenotyping of aberrant plasma cells.^2, 6, 7, 8, 9, 10^

Recently, interest in the use of imaging modalities other than bone surveys to risk stratify patients with SMM has emerged. Hillengass *et al.*^11^ found that the presence of one or more focal lesion on magnetic resonance imaging in patients with SMM was a strong predictor of progression to active MM. They also found that progression on magnetic resonance imaging was associated with a high probability of progression to MM, regardless of the initial magnetic resonance imaging finding.^12^

Limited data are available on the use of 18F-fluorodeoxyglucose positron emission tomography combined with computerized tomography (PET-CT) to guide decision making in SMM. We hypothesize that PET-CT imaging at the time of SMM diagnosis can be used to identify patients at high risk of disease progression within 2 years.

## Materials and methods

We identified all patients with a diagnosis of SMM (based on standard disease definition prior to recent updated IMWG criteria) from January 2000 to March 2014 who had undergone a PET-CT scan as part of their clinical evaluation by using the Mayo Clinic Data Discovery and Query Database and a review of available medical records. The PET-CT findings, results of other diagnostic tests and clinical course were then abstracted. A positive PET-CT was defined as a radiologist interpretation of abnormal increased uptake (diffuse and/or one or more focal skeletal areas) and/or evidence of lytic bone destruction on the CT portion of the exam. The primary end point was progression to active MM within the first 2 years following a positive PET-CT result among patients observed without therapy. Secondary end points included the proportion of patients in whom the diagnosis of active MM was made based solely on the findings of the PET-CT, the probability of progression within 2 years in patients with a negative PET-CT who were observed without therapy, and estimating differences in the probability of progression based on the presence or absence of underlying osteolysis in patients with a positive PET-CT.

The *χ*^2^ test was used to compare nominal values. Time to progression (TTP) was measured from the date of PET-CT until progression to active MM. Kaplan–Meier analysis was performed to generate progression and survival curves. Time to event and survival between groups was compared with the two-tailed log-rank test.

## Results

**Figure 1:**
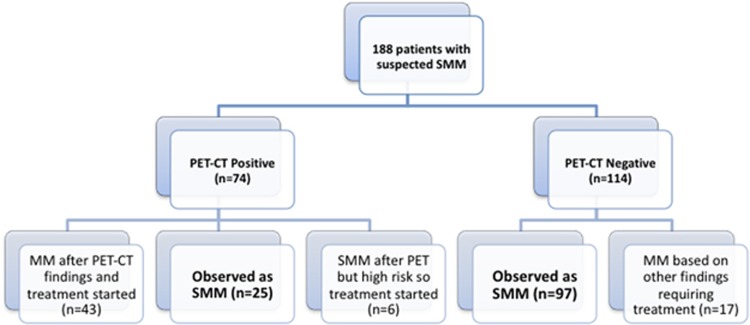
Disposition of patients. PET-CT, positron emission tomography-computerized tomography; SMM, smoldering multiple myeloma.

One hundred and eighty-eight patients were identified with a suspected diagnosis of SMM in whom a PET-CT scan had been performed as part of the diagnostic evaluation. PET-CT was positive in 74 patients, and negative in 114 patients. Of the 74 patients with a positive PET-CT, 49 were diagnosed and treated as MM, whereas 25 were considered to still have SMM and observed (Figure 1). Of the 49 patients diagnosed as MM, 12 (24%) were upstaged to the diagnosis of active disease solely based on the findings of the PET-CT; in the remaining 37 patients, myeloma defining events were identified on other laboratory tests conducted during the same visit. Similarly, of the 114 patients with a negative PET-CT, 17 (14%) were diagnosed and treated as MM based on other laboratory parameters, whereas 97 were considered to have SMM and observed. Thus, 25 patients with a positive PET-CT and 97 patients with a negative PET-CT who were observed without therapy formed the principal cohort (*n*=122) for this study (Figure 1). Patient characteristics are provided in Table 1. The rate of progression to MM within 2 years was then compared between these two groups.

**Figure 2:**
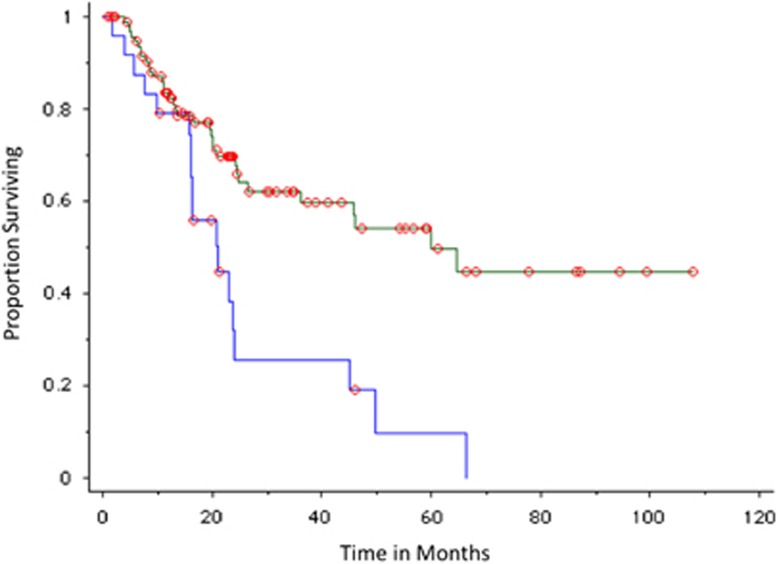
TTP of SMM in patients observed without therapy, including all patients with PET-CT imaging. Blue curves represent positive (abnormal PET-CT) imaging result; green curves represent negative imaging results.

The probability of progression to MM within 2 years was 75% in patients with a positive PET-CT (*n*=25) compared with 30% in patients with a negative PET-CT (*n*=97); median TTP was 21 months versus 60 months, respectively, *P*=0.0008 (Figure 2). The mode of progression in patients with a positive PET-CT who progressed was anemia (10 patients, including one patient who also developed amyloidosis and one patient with central nervous system disease), bone disease (6 patients, including 2 patients with concurrent renal failure and one with cryoglobulinemia), renal failure (1 patient) and rapid rise in M protein (1 patient).

**Figure 3:**
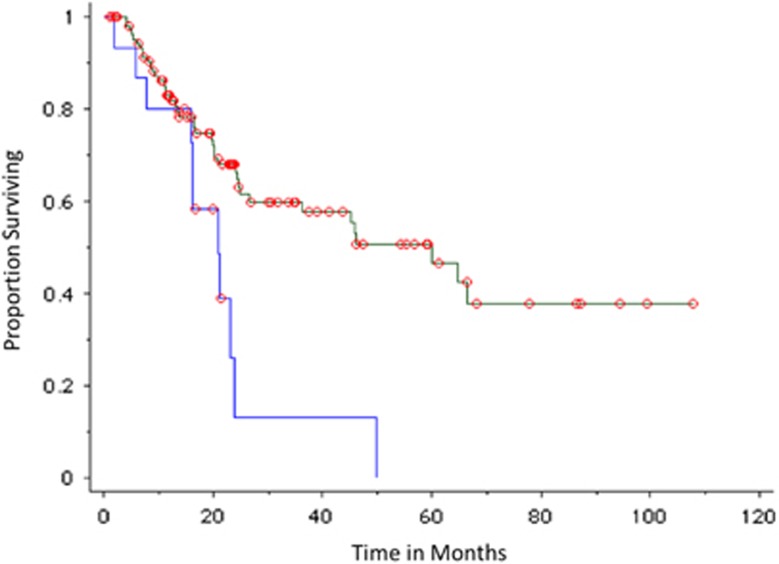
TTP of SMM in patients observed without therapy based on osteolysis on PET-CT. Blue curves represent positive (abnormal bone/osteolytic lesion present) imaging result; green curves represent negative (no bone abnormality) imaging results.

The median TTP in patients with positive PET-CT and underlying osteolysis (*n*=16) was 21 months compared with 60 months in patients with no evidence of osteolysis on PET-CT (*n*=106), *P*=0.004 (Figure 3). Among patients with a positive PET-CT, the probability of progression was 87% at 2 years in the subset of patients with underlying osteolysis (*n*=16) and 61% in patients with abnormal PET-CT uptake but no evidence of osteolysis (*n*=9), *P*=0.31.

**Figure 4:**
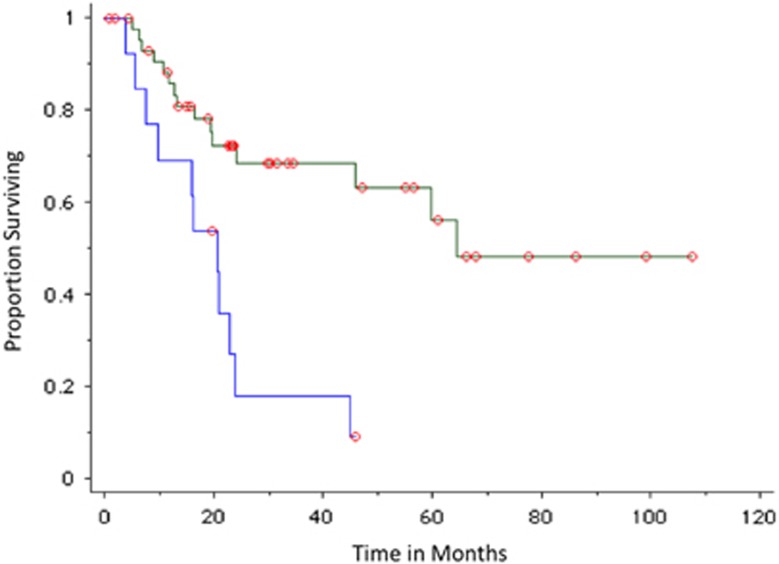
TTP of SMM in patients observed without therapy based on PET-CT imaging carried out within 90 days of diagnosis. Blue curves represent positive (abnormal PET-CT) imaging result; green curves represent negative imaging results.

Analysis was then restricted to 59 patients in whom the PET-CT was carried out within 90 days of diagnosis of SMM. The probability of progression to MM within 2 years was 82% in patients with a positive PET-CT (*n*=13) compared with 28% in patients with a negative PET-CT (*n*=46); median TTP in patients with a positive PET-CT was 21 months compared with 65 months in patients with a negative PET-CT, *P*=0.0006 (Figure 4).

**Figure 5:**
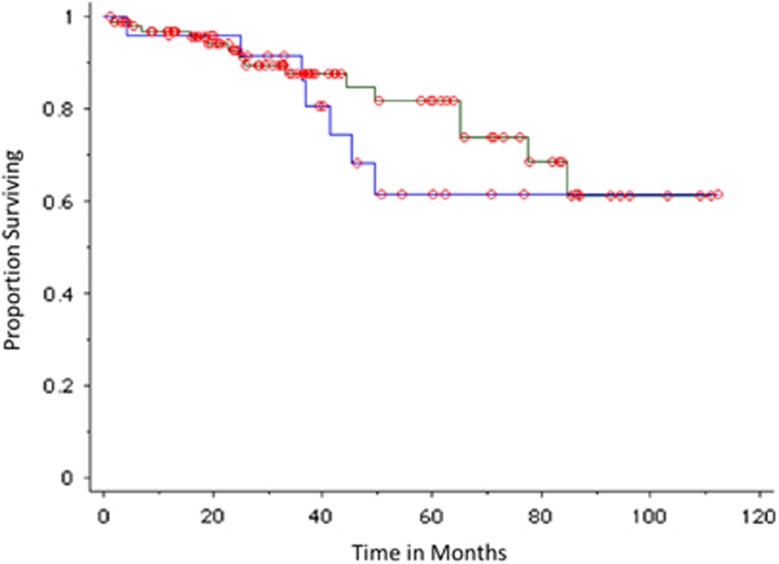
Overall survival of SMM patients based on PET-CT imaging results. Blue curve represents positive (abnormal) imaging results; green curve represents negative imaging results.

Median overall survival was not reached. The 5-year survival rate was 61% versus 82% in patients with positive and negative PET-CT, respectively, but the difference was not statistically significant, *P*=0.42 (Figure 5).

Thirteen patients had a serum FLC ratio of ⩾100; of these, only three had a positive PET-CT and two had disease progression (both at 16 months). Five patients had a BMPC ⩾60% none of these patients had a positive PET-CT. Only one patient had both serum FLC ratio of ⩾100 and BMPC ⩾60%. The probability of progression to MM in patients with a positive PET-CT remained unchanged when analysis was repeated after excluding the 17 patients who had either serum FLC ratio of ⩾100 or BMPC ⩾60%. The probability of progression to MM within 2 years was 77% in patients with a positive PET-CT (*n*=22) compared with 31% in patients with a negative PET-CT (*n*=83); median TTP was 21 months versus not reached, respectively, *P*=0.0002.

## Discussion

SMM is a plasma cell proliferative disorder with a risk of progression to MM of ~10% per year in the first 5 years following diagnosis, 3% per year subsequently for the next 5 years and ~1.5% per year thereafter.^2^ While the current standard of care remains observation, concern exists that patients at high risk of progression can sustain unacceptable end-organ damage despite careful observation.^13^ Based on specific biomarkers, the IMWG recently reclassified as MM a small proportion of patients with SMM and ultra-high risk of progression (~40% per year in the first 2 years).^3^ The IMWG also clarified that evidence of osteolysis on CT or PET-CT meets the definition of bone disease in MM. This recommendation was supported by a systematic review that compared newer imaging modalities, magnetic resonance imaging, 18F-fluorodeoxyglucose positron emission tomography, PET-CT and whole-body CT, to conventional whole-body skeletal radiography^14^ and found that newer imaging techniques had greater sensitivity compared with radiographic bone survey for the detection of MM bone lesions.

In clinical practice, patients with osteolytic lesions visible on CT or PET-CT but not seen on skeletal radiography have often been treated as MM even before the updated IMWG criteria were published. Coupled with the relative rarity of SMM, studies of the natural history of such patients based on the results of PET-CT imaging is scarce. In this study, we show that patients with suspected SMM who have an abnormal PET-CT (clinical radiologist interpretation of increased bone uptake and/or evidence of lytic bone destruction) who are observed without therapy are at high risk (75%) of progression to MM within 2 years. This risk increases to 87% in patients with evidence of underlying osteolysis on PET-CT. Importantly, these estimates likely underestimate the true risk of progression to myeloma as they exclude patients with presumably higher grade lesions on PET-CT who were initiated on therapy based solely on the PET-CT finding (*n*=12 in this cohort).

We found PET-CT identifies patients at high risk of progression independent of the new serum FLC and BMPC thresholds established to define MM in the revised IMWG criteria. Only three patients with those ultra-high-risk biomarkers (serum FLC ratio ⩾100 or BMPC ⩾60%) had a positive PET-CT in our study. The probability of progression to MM was not affected when patients meeting new IMWG criteria based on the FLC assay or BMPC% were excluded from the analysis.

Our study also found that patients with increased focal uptake but no evidence of osteolysis are also at high risk of progression to myeloma, with a 61% risk of progression within 2 years. This is similar to a report by Zamagni *et al.*^15^ who found that 12% of patients with SMM have increased focal uptake on PET-CT without underlying osteolysis. The probability of progression within 2 and 3 years for such patients (*n*=9) was 48% and 65%, respectively, in comparison with 32% and 42% for patients with a negative PET-CT (*n*=64).^15^ Patients with increased focal uptake without underlying osteolysis should be considered to have high-risk SMM and need close observation and consideration of clinical trials testing prophylactic therapy.^1^

In summary, our study results align with the recently updated IMWG criteria for the diagnosis of MM. Patients with suspected SMM who have evidence of clear osteolytic bone destruction on PET-CT that is attributable to the underlying plasma cell disorder should be considered to have MM.

## Figures and Tables

**Table 1:** Baseline characteristics

	*Patients*, N=*122*
Median age, years (range)	69 (35–92)
Female sex, *N* (%)	61 (50%)
Serum monoclonal protein spike, median (range) (g/dl)	2.0 (0.0–4.6)
	
*Urine monoclonal protein spike (*n=*96),* N *(%)*	
Not present	29 (30)
Detected on immunofixation only	39 (41)
Measurable but <0.5 g per 24 h	24 (25)
0.5 g per 24 h or more	3 (3)
	
Bone marrow plasma cell percentage, median (range)	20 (4.0–100)
*Serum-free light-chain assay (*n=*91),* N *(%)*	
Abnormal κ/λ FLC ratio (<0.26 or >1.65)	12 (13)
Abnormal involved/uninvolved FLC ratio ⩾8	57 (63)
Abnormal involved/uninvolved FLC ratio ⩾100	13 (14)

Abbreviation: FLC, free light chain.
